# New Methods for the Comprehensive Analysis of Bioactive Compounds in *Cannabis sativa* L. (hemp)

**DOI:** 10.3390/molecules23102639

**Published:** 2018-10-14

**Authors:** Federica Pellati, Virginia Brighenti, Johanna Sperlea, Lucia Marchetti, Davide Bertelli, Stefania Benvenuti

**Affiliations:** 1 Department of Life Sciences, University of Modena and Reggio Emilia, Via G. Campi 103, 41125 Modena, Italy; virginia.brighenti@unimore.it (V.B.); johanna.sperlea@ernaehrung.uni-giessen.de (J.S.); lucia.marchetti@unimore.it (L.M.); davide.bertelli@unimore.it (D.B.); stefania.benvenuti@unimore.it (S.B.); 2 Faculty of Agricultural Sciences, Nutritional Sciences, and Environmental Management, Justus-Liebig University of Giessen, Goethestrasse 58, 35390 Giessen, Germany

**Keywords:** *Cannabis sativa* L., hemp, cannabinoids, flavonoids, terpenes, HPLC, GC, MS.

## Abstract

*Cannabis sativa* L. is a dioecious plant belonging to the *Cannabaceae* family. The main phytochemicals that are found in this plant are represented by cannabinoids, flavones, and terpenes. Some biological activities of cannabinoids are known to be enhanced by the presence of terpenes and flavonoids in the extracts, due to a synergistic action. In the light of all the above, the present study was aimed at the multi-component analysis of the bioactive compounds present in fibre-type *C. sativa* (hemp) inflorescences of different varieties by means of innovative HPLC and GC methods. In particular, the profiling of non-psychoactive cannabinoids was carried out by means of HPLC-UV/DAD, ESI-MS, and MS^2^. The content of prenylated flavones in hemp extracts, including cannflavins A and B, was also evaluated by HPLC. The study on *Cannabis* volatile compounds was performed by developing a new method based on headspace solid-phase microextraction (HS-SPME) coupled with GC-MS and GC-FID. Cannabidiolic acid (CBDA) and cannabidiol (CBD) were found to be the most abundant cannabinoids in the hemp samples analysed, while β-myrcene and β-caryophyllene were the major terpenes. As regards flavonoids, cannflavin A was observed to be the main compound in almost all the samples. The methods developed in this work are suitable for the comprehensive chemical analysis of both hemp plant material and related pharmaceutical or nutraceutical products in order to ensure their quality, efficacy, and safety.

## 1. Introduction

In recent years, *Cannabis sativa* L. has been the focus of the attention of the scientific community all over the world, definitely becoming one of the most studied plants [1]. It is one of the two representatives of the *Cannabaceae* family and nowadays it is widely distributed all over the world. *C. sativa* has been cultivated for a long time for medicinal, food, and abuse purposes, together with its use as a source of textile fibre [1,2].

The taxonomy of the plant has always represented a critical issue, due to the huge variability within the same genus [1,2,3]. Recently, a monotypic classification has been preferred, in which one species (*C. sativa*) is recognised and it is divided into different chemotypes on the basis of the specific cannabinoid profile [3]. Nonetheless, in the pharmaceutical field, only two phenotypes are considered, on the basis of the content in the psychoactive principle Δ^9^-tetrahydrocannabinol (Δ^9^-THC) [4]. In detail, the first one is drug-type *Cannabis*, which is rich in psychoactive Δ^9^-THC, and is used for medicinal or recreational purposes [4]; the second one is fibre-type *Cannabis* (commonly known as hemp or industrial hemp), used for both textile or food purposes, which has content of Δ^9^-THC below the legal limit of 0.2–0.3% [2,5] and is rich in non-psychoactive cannabinoids. Currently, 69 hemp varieties have been approved for commercial use by the European Community [6]. 

The pharmaceutical interest in this plant has been mainly addressed to drug-type *Cannabis*, thanks to its new therapeutic applications [7], while hemp is at the moment under-employed in this ambit. However, the number of studies focused on the characterisation of fibre-type hemp varieties and on the evaluation of the biological potential of non-psychoactive compounds has increased lately [2,3,8,9,10,11,12,13,14,15,16].

**Figure 1:**
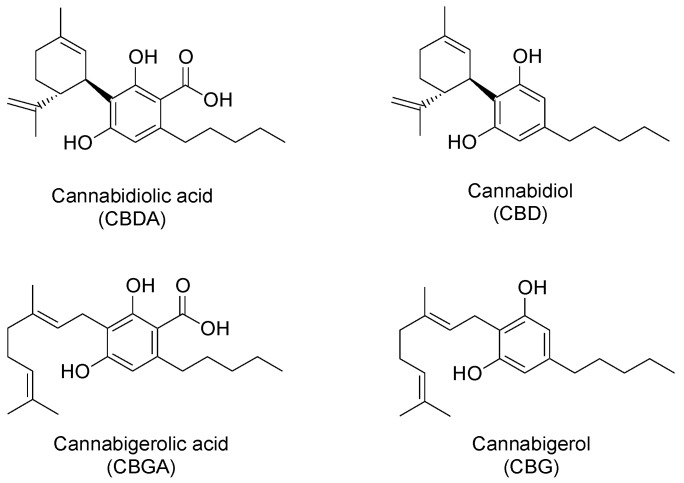
Chemical structures of main non-psychoactive cannabinoids in hemp inflorescences.

The chemistry of *C. sativa* is known to be very complex; indeed, several chemical classes have been identified in the plant, encompassing terpenes, carbohydrates, fatty acids and their esters, amides, amines, phytosteros, phenolic compounds, and cannabinoids [17]. From a chemical point of view, the latter are meroterpenoids and they are specific of this plant [2,17]. Usually, the most abundant cannabinoids present in fibre-type plants are cannabinoic acids, such as cannabidiolic acid (CBDA) and cannabigerolic acid (CBGA), followed by their decarboxylated forms, namely cannabidiol (CBD) and cannabigerol (CBG) (Figure 1) [9]. In plant tissues, cannabinoids are biosynthesized in the acid form; environmental factors, such as heat and light, generally induce a spontaneous decarboxylation process to form decarboxylated cannabinoids [2,4,9,16,18,19]. 

Among non-psychoactive cannabinoids, CBD represents the most promising one from the pharmaceutical point of view, due to its high anti-oxidant and anti-inflammatory activity, in addition to its anticonvulsant, anxiolytic, neuroprotective, and antibiotic properties [2,4,20,21,22,23]. CBDA has antimicrobial and anti-nausea properties [2,20,22], while CBG has anti-inflammatory, antimicrobial and analgesic activities [2,20,22,24]. 

**Figure 2:**
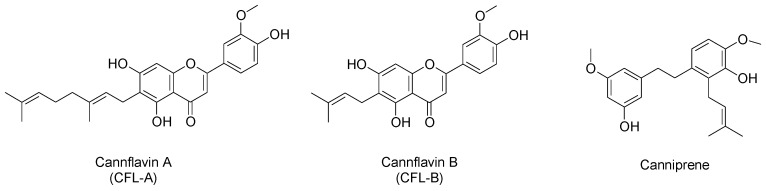
Chemical structures of main flavonoids and related compounds in hemp inflorescences.

Concerning other phenolic compounds, several flavonoids have been identified in *C. sativa*, belonging mainly to flavones and flavonols [25]. In particular, cannflavin A and B represent hemp-specific methylated isoprenoid flavones (Figure 2) [25]. Several biological effects have been ascribed to hemp flavonoids, including anti-inflammatory, anti-microbial, neuroprotective, and anti-proliferative activities [17]. In particular, cannflavin A and B are known to possess an anti-inflammatory action [17] and microsomal prostaglandin E_2_ synthase (mPGES-1) and 5-lipoxygenase (5-LO) have been identified as their molecular targets [26]. In addition to flavonoids, dihydrostilbenoids represent another class of polyphenolic substances isolated from hemp, of which canniprene is the main representative (Figure 2) [25]. As to canniprene, it has been demonstrated to exert an anti-inflammatory activity as well, by inhibiting the production of pro-inflammatory eicosanoids [8]. 

**Figure 3:**
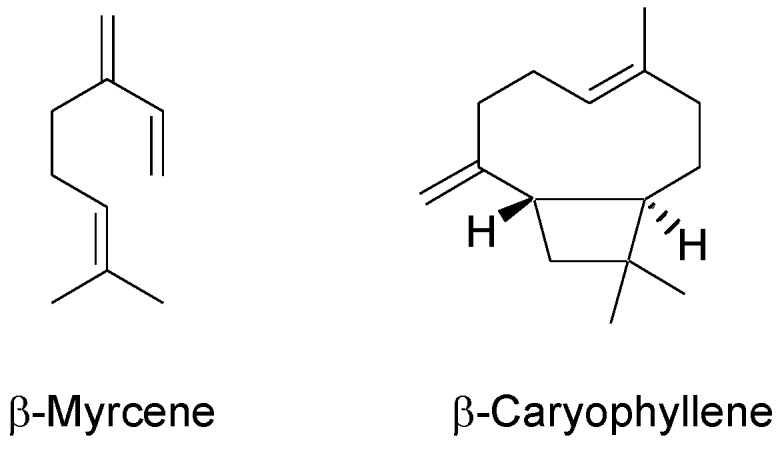
Chemical structures of main terpenes in hemp inflorescences.

As regards the other compounds present in hemp, terpenes are responsible for the characteristic aroma of the plant. The main volatiles detected in the aerial parts of the plant include both mono- and sesquiterpenes [14,15,17,27,28,29], with β-myrcene and β-caryophyllene as the most representative compounds (Figure 3), respectively. As regards monoterpenes, β-myrcene is known to possess anti-inflammatory, analgesic, and anxiolytic properties [17]. As for sesquiterpenes, β-caryophyllene was found to be an anti-inflammatory agent and to exert a gastric cytoprotector activity; it has been demonstrated also to be able to bind to the cannabinoid receptors type 2 (CB_2_) and, in this context, it can be considered as a phytocannabinoid [17].

Some biological activities of cannabinoids are known to be enhanced by the presence of other secondary metabolites in *C. sativa* extracts, such as in cases of sleeping disorders and anxiety [30]. This effect has been attributed to a strict interaction between cannabinoids and terpenes, resulting in a synergistic action [30]. As an example, terpenes are able to increase blood-brain barrier permeability and they can also interact with neurotransmitter receptors, thus contributing to cannabinoid-mediated analgesic and psychotic effects [17]. Finally, also flavonoids may modulate the pharmacokinetics of some cannabinoids, by means of the inhibition of hepatic P450 enzymes [17].

With this in view, the development and application of advanced analytical methods for a comprehensive analysis of *C. sativa* extracts plays a pivotal role in order to have a reliable evaluation of their chemical composition both for a higher reproducibility of biological assays and also to guarantee the efficacy and safety in their pharmaceutical use. 

In the light of all the above, the present study was aimed at the first multi-component analysis of the bioactive compounds present in hemp female inflorescences of different varieties, including cannabinoids, hemp-specific phenolics, and volatile compounds. Innovative HPLC and GC methods were developed and applied in this study to the chemical classes of compounds cited above. In particular, the profiling of non-psychoactive cannabinoids in hemp extracts was carried out by means of HPLC with UV/DAD, ESI-MS, and ESI-MS^2^ detection. A new RP-HPLC-UV/DAD, ESI-MS, and ESI-MS^2^ method, together with a selective extraction protocol, was developed as well and applied for the determination of hemp phenolics (including cannflavin A, cannflavin B, and canniprene). The study on *C. sativa* volatile compounds was performed by developing a new method based on headspace solid-phase microextraction (HS-SPME) coupled with GC-MS and GC-FID. The analytical methods developed in this study represent reliable and useful tools for a complete characterization of hemp plant material and extracts to be used in the pharmaceutical and nutraceutical fields.

## 2. Results and Discussion

### 2.1. Extraction of Non-Psychoactive Cannabinoids and Flavonoids from Hemp Inflorescences

The development and application of a suitable extraction procedure covers a crucial role to achieve a reliable characterization of a plant material. For what concerns cannabinoids, a simple dynamic maceration (DM) at room temperature was applied by using ethanol (EtOH) as the extraction solvent, which has been demonstrated to be the best solvent for the extraction of these bioactive compounds [9]. EtOH is indeed a good solvent for the extraction of many phenolics from plant material [31]. As a matter of fact, cannflavins A and B were detected in hemp ethanolic extracts as well, but, conversely to cannabinoids, their content in the inflorescences was extremely low [8,10,25]; in addition, the concomitant presence of cannabinoids in the same extract made them difficult to be quantified. For this reason, two different protocols were optimised and applied in this work for the extraction of non-psychoactive cannabinoids and flavonoids from hemp.

**Figure 4:**
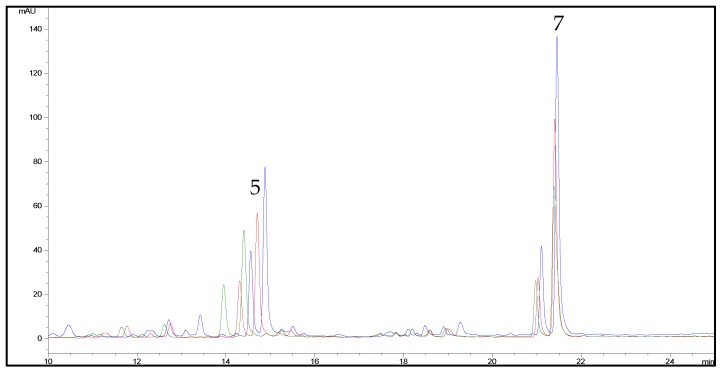
HPLC-UV/DAD chromatograms of a hemp extract (sample C6) in acetone (blue line), EtOAc (red line) and EtOH (green line), recorded at 342 nm. For peak numbering, see Table 1.

Starting with the extraction conditions previously optimised for cannabinoids, the method was modified as follows. First, the solvent was properly chosen in order to have a good yield of hemp flavonoids from the plant material; to do this EtOH, acetone and ethyl acetate (EtOAc) were tested. The extracts were also concentrated 25:1 before HPLC analysis, in order to increase the intensity of the UV/DAD signal. Of the solvents tested, acetone resulted in being the best one and it was therefore selected for use in the subsequent steps (Figure 4).

**Figure 5:**
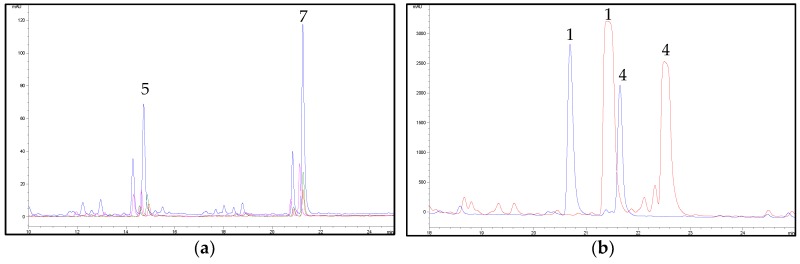
(**a**) Effect of different pre-maceration procedures on flavonoid extraction from hemp (sample C6). Chromatograms obtained at 342 nm with pre-maceration with *n-*hexane (3x) (blue line), with *n-*hexane (2x) + dichloromethane (DCM) (1x) (red line), with *n-*hexane (2x) + ethyl ether (1x) (green line), with *n-*hexane (2x) + toluene (1x) (pink line). For peak numbering, see Table 1. (**b**) Effect of pre-maceration with *n*-hexane on cannabinoid removal from hemp (sample C6). Chromatograms obtained at 210 nm with pre-maceration with *n*-hexane (3x) (blue line) and without pre-maceration (red line). For peak numbering, see Table 1.

In order to reduce any possible interference from cannabinoids in the concentrated extract, a sample pre-treatment was considered necessary. Indeed, the amount of cannabinoids in hemp inflorescences is much higher than that of flavonoids; thus, the peaks related to these compounds in the extracts may interfere with the quantification of other constituents at 210 nm. Conversely to other phenolics, cannabinoids can dissolve also in non-polar solvents (i.g. *n*-hexane, chloroform, dichloromethane (DCM)) [9]; hence, a sample pre-treatment by means of DM repeated three times with a non-polar solvent, including *n*-hexane and its combination with DCM, ethyl ether and toluene, was tested to be suitable to remove cannabinoids. After this treatment, the residue was extracted three times with acetone by means of DM. As shown in Figure 5, *n*-hexane alone resulted in being the most suitable solvent for the sample pre-maceration in comparison with other non-polar solvents tested. Indeed, its combination with DCM or ethyl ether or toluene, led to a decrease in the final amount of flavonoids extracted from hemp. 

**Figure 6:**
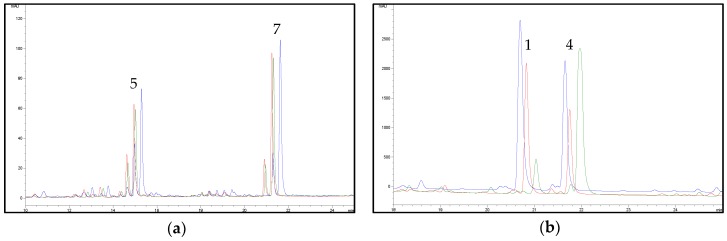
(**a**) Effect of different thermal treatments on flavonoid extraction from hemp (sample C6). Chromatograms obtained at 342 nm without thermal treatment (blue line), with thermal treatment at 80 °C for 2 h (red line), with thermal treatment at 120 °C for 1 h (green line). For peak numbering, see Table 1. (**b**) Effect of different thermal treatments on cannabinoid removal from hemp (sample C6). Chromatograms obtained at 210 nm without thermal treatment (blue line), with thermal treatment at 80 °C for 2 h (red line), with thermal treatment at 120 °C for 1 h (green line). For peak numbering, see Table 1.

Finally, in order to facilitate the removal of cannabinoids, a decarboxylation of the plant material, prior to the pre-maceration with *n*-hexane, was also taken into account in order to convert cannabinoic acids into their neutral counterparts, which are more soluble in this solvent. In particular, the plant material was heated at 120 °C for 1 h or at 80 °C for 2 h, respectively. After this decarboxylation step, the pre-maceration process with *n*-hexane and the extraction with acetone previously optimised were applied. As shown in Figure 6**,** the peak areas related to flavonoids decreased after the heating process and the removal of cannabinoids was not significantly increased; therefore, it was not further pursued. In the light of all the above, a two-step extraction, based on an initial pre-maceration of the plant material with *n*-hexane and on the subsequent extraction with acetone, was selected as the best protocol for hemp flavonoids.

The optimized extraction techniques, based on dynamic maceration at room temperature, did not cause the degradation of the compounds of interest or the formation of artefacts.

### 2.2. HPLC Methods for the Analysis of Non-Psychoactive Cannabinoids and Flavonoids in Hemp Extracts

The aim of the present study was to provide a comprehensive analysis of the main bioactive compounds in fibre-type *C. sativa* inflorescences. In this perspective, the development of HPLC methods for the determination of non-psychoactive cannabinoids and hemp-specific phenolics was a critical aspect of the research. RP-HPLC on C_18_ columns, with acidic methanol (MeOH) or acetonitrile (ACN) used as the strong solvent eluent and acidic H_2_O as the weak one, is frequently used for the analysis of cannabinoids in *C. sativa* extracts [3,9,13,16]. Indeed, the use of an acidic mobile phase improves the separation performance, providing better peak shape and enhanced resolution, along with the enhancement of their ionization in HPLC-ESI-MS and MS^2^ experiments [3,9,13]. For acidification, HCOOH is usually employed [3,9,13]. For our purpose, an Ascentis Express C_18_ (150 × 3.0 mm I.D., 2.7 µm, Supelco) as the chromatographic column and a gradient mobile phase composed of H_2_O and ACN, both containing 0.1% of HCOOH were selected, on the basis of a previous work [9]. 

**Figure 7:**
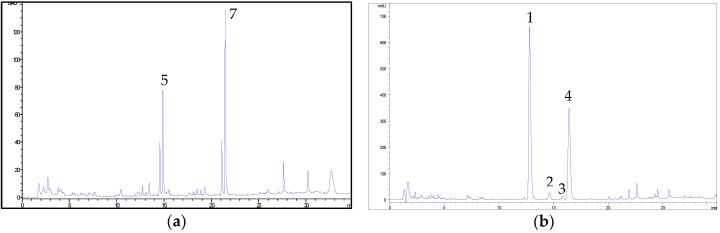
(**a**) Representative HPLC-UV/DAD chromatogram of a hemp extract (sample C6) at 342 nm obtained by using the analytical method optimised for flavonoids and related compounds. For peak numbering, see Table 1. (**b**) Representative HPLC-UV/DAD chromatogram of a hemp extract (sample C6) at 210 nm obtained by using the analytical method optimised for cannabinoids. For peak numbering, see Table 1.

The gradient elution with the same mobile phase was then properly modified for hemp flavonoids, since peaks corresponding to cannflavins A and B were observed to elute with the solvent front. Under the final conditions, a good resolution of peaks belonging to cannflavins A and B and other hemp phenolics was achieved. Representative chromatograms obtained by using the two HPLC methods are shown in Figure 7. 

### 2.3. Identification of Non-Psychoactive Cannabinoids and Flavonoids in Hemp Extracts

The identification of secondary metabolites in hemp extracts was carried out by combining the information obtained from UV/DAD and MS detectors. For a complete characterisation, the experimental data were compared with the literature [3,9,12,32] and with commercially available reference standards.

The UV spectra were used to preliminarily assign the chromatographic peaks to a chemical class. As a matter of fact, cannabinoic acids, such as CBDA and CBGA, can be easily distinguished from their decarboxylated counterparts (CBD and CBG, respectively) by their UV/Vis spectra: CBDA and CBGA are indeed characterised by three absorption maxima (λ_max_), one at 220–223 nm, a second one at 266–270 nm and a third one around 305 nm; CBD and CBG show a first λ_max_ at 210–215 nm and an additional one at 270 nm [9,13]. Canniprene showed a UV/Vis spectrum comparable to that of neutral cannabinoids, with a λ_max_ at 204 nm and a minor one at 280 nm. Also cannflavins can be recognised on the basis of their UV/Vis spectra, since they possess a representative chromophore: indeed, the spectra of cannflavins A and B showed a first λ_max_ at 213–215 nm, a second one at around 273–274 nm and, finally, one at 342 nm. 

As regards MS and MS^2^, the experiments were carried out both in the positive and in the negative ion mode. The MS and MS^2^ data are shown in Table 1. In particular, cannabinoic acids showed a higher intensity of the signals in the negative ion-mode, as opposed to decarboxylated compounds and canniprene, which ionised better in the positive ion mode. Conversely to cannabinoids, flavonoids (cannflavin A and B) were only detectable when positively ionised. 

As regards the MS data acquired in the positive ion mode, both CBDA and CBGA generated sodium adduct ions [M + Na]^+^ [3], in addition to their pseudo-molecular ions [M + H]^+^. CBGA was easily distinguished from CBDA, thanks to its different molecular weight. As to their MS^2^ spectra carried out in the positive ion mode, both CBDA and CBGA showed base peaks related to the loss of water (−18 u) at *m*/*z* 341 and 343, respectively. CBDA showed an additional product ion at *m*/*z* 285, attributable to the loss of a C_4_H_8_ group [9,33], which was not observed in the MS^2^ spectrum of CBGA. Both CBG and CBD showed product ions at *m*/*z* 233 and 207 in the MS^2^ experiments performed in the positive ion mode, although their relative intensity was different. The product ion at *m*/*z* 259 in the MS^2^ spectrum of CBD was due to the loss of a C_4_H_8_ group [9,33], probably from the side alkyl chain, which was absent in the MS^2^ spectrum of CBG. As to canniprene, the MS spectrum carried out in the positive ion mode showed the pseudo-molecular ion [M+H]^+^ at *m*/*z* 343 and a base peak at *m*/*z* 287, due to the loss of a C_4_H_8_ group of the side prenyl group. In the MS spectra of cannflavins A and B only the pseudo-molecular ion [M + H]^+^ was observed. The MS^2^ spectra of these compounds showed only one intense fragment at *m*/*z* 313, generated after the loss of the side prenyl moiety. 

As for the MS data obtained in the negative ion mode, both CBDA and CBGA generated product ions corresponding to the loss of water (−18 u) and of CO_2_ (−44 u), together with their pseudo-molecular ions [M − H]^−^. Concerning the MS^2^ spectra acquired in the negative ion-mode, CBG generated a product ion at *m*/*z* 247, due to the loss of a C_5_H_8_ group; additional fragments were observed at *m*/*z* 297, 271, and 204. The fragmentation of CBD in the negative ion-mode provided almost exclusively one product ion at *m*/*z* 245, attributable to the loss of a C_5_H_8_ group, following a retro Diels-Alder reaction, which is a common fragmentation pathway in the field of natural substances when an ESI ion source is employed [9,34]. Canniprene generated its pseudo-molecular ion [M − H]^−^ at *m*/*z* 341 in the negative ion mode; its MS^2^ spectrum showed a major product ion at *m*/*z* 326, attributable to the loss of a methyl group. 

It should be specified that the two peaks that precede each cannflavin belong to different flavonoids, which have not been identified. Even though the UV/Vis spectrum was observed to resemble those of flavones, the molecular mass and the fragmentation pattern was found to be different in the MS and MS^2^ experiments. 

### 2.4. Validation Data of the HPLC Methods 

Over the concentration range tested, the two HPLC-UV/DAD methods showed good linearity (*r*^2^ > 0.999) for the reference standards chosen in this study (Table S1).

The limit of detection (LOD) values for chrysoeriol and canniprene were 0.4 and 0.1 µg/mL, respectively, while for cannabinoids it ranged from 0.4 and 0.8 µg/mL. The limit of quantification (LOQ) values obtained for cannflavins and canniprene were 1.3 and 0.3 µg/mL, respectively, while for cannabinoids it was in the range 1.3–2.5 µg/mL (Table S1). 

The low intra- and inter-day % relative standard deviation (%RSD) for retention times (≤2.4%) and peak areas (≤3.2%) relative to the target compounds (Table S2) and their low intra- and inter-day SD values for content (≤21.3 µg/g) (Table S3) indicate the high precision of both the chromatographic system and the extraction procedure. 

By taking into account all the information described above, it can be concluded that this method is a reliable tool for the identification and quantification of flavonoids in fibre-type hemp, conforming to the ICH guidelines.

### 2.5. Quantitative Analysis of Non-Psychoactive Cannabinoids in Hemp by HPLC-UV/DAD 

Quantitative data related to the content of non-psychoactive cannabinoids in hemp inflorescences determined by means of the HPLC-UV/DAD method are shown in Table 2. Since all the samples analysed are fibre-type hemp varieties, it is not surprising that the most abundant cannabinoids were represented by CBDA and CBD [9]. In particular, samples C1, C3, and C6 showed the typical cannabinoid profiles of the fresh plant material, with CBDA as the main compound, followed by CBD. On the other hand, samples C2, C4, and C5 showed the opposite behaviour: as a matter of fact, CBD represented the most abundant cannabinoid in these samples, followed by CBDA. This profile may be attributed either to an aged plant material or to a non-optimal drying process applied to the inflorescences or to unsuitable storage conditions that may have led to a partial conversion of the parent cannabinoic acid into its neutral counterpart, via a decarboxylation process, which naturally occurs with time under the action of heat and light [9]. 

The same trend was observed also for the other two major non-psychoactive cannabinoids, namely CBGA and CBG, though their content was about 10 times lower in comparison to CBDA and CBD and, in some cases, not quantifiable. Nevertheless, the amount of non-psychoactive cannabinoids determined in the analysed samples is in agreement with what is described in the literature for the content of cannabinoids in hemp female inflorescences [9]. 

### 2.6. Quantitative Analysis of Flavonoids and Related Compounds in Hemp by HPLC-UV/DAD 

The content of the two flavonoids cannflavin A and B and the stilbenoid canniprene in the hemp samples analysed in this work is shown in Table 3. Canniprene was not detected in any of the samples analysed, even though its presence has been previously reported in the literature for fibre-type hemp varieties, comprising C2 and C3 [8]. However, Allegrone et al. [8] observed that a huge variation in the amount of canniprene in hemp inflorescences may occur; this can explain why this compound was not detected in any of the samples analysed in this study [8].

As regards flavonoids, cannflavin A was observed to be the most abundant one in all the samples, with the only exception of sample C3, which had a high content of both cannflavins A and B. These quantification data are in good agreement with the existing literature [8,10,25].

### 2.7. Extraction of Volatile Compounds from Hemp by HS-SPME 

The terpene composition of hemp inflorescences has been found to enhance the biological activity of hemp-based products [30]; in this perspective, the development of a fast and reliable procedure for the extraction of the volatile compounds from the plant material is highly recommended for its complete characterisation. 

HS-SPME is being increasingly selected as the technique of choice in the ambit of the extraction of volatile compounds in the field of natural product research [14,15,27,28]. In this study, a new HS-SPME procedure was optimised in order to achieve a reliable characterisation of the volatiles from hemp inflorescences. To do this, sample C3 was used for the optimisation of the HS-SPME conditions, as the essential oil from the same variety was also available. 

**Figure 8:**
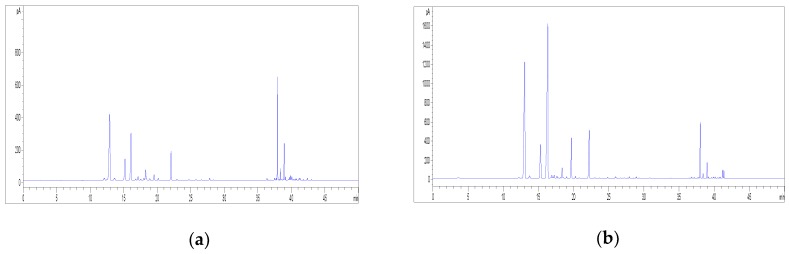
Effect of fibre type on the HS-SPME-GC-FID profiles of hemp inflorescences (sample C3): (**a**) PDMS fibre; (**b**) DVB/CAR/PDMS fibre.

Firstly, the performance of two different SPME fibres was assessed, including both a polydimethylsiloxane (PDMS) and a divinylbenzene-carboxen-polydimethylsiloxane (DVB/CAR/PDMS) fibre. The representative GC-FID chromatograms of the HS profile of hemp inflorescences obtained by using the PDMS and the DVB/CAR/PDMS fibres are shown in Figure 8. In order to compare the fibre performance, the same temperature, equilibrium time, and extraction time were employed. The extraction efficiency of volatile compounds was found to be higher when the DVB/CAR/PDMS fibre was used; this fibre was therefore selected for this work.

**Figure 9:**
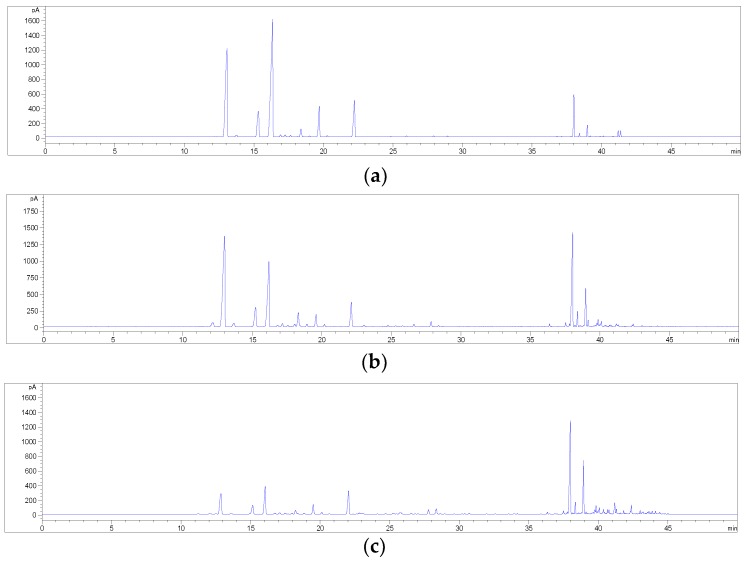
Effect of temperature on the HS-SPME-GC-FID profiles of hemp inflorescences (sample C3) with the DVB/CAR/PDMS fibre: (**a**) 40 °C; (**b**) 60 °C; (**c**) 80 °C.

**Figure 10:**
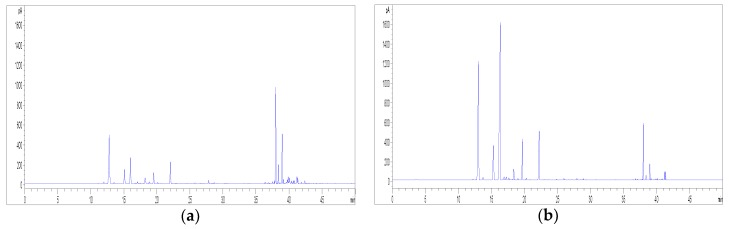
Effect of the equilibrium time and extraction time on the HS-SPME-GC-FID profiles of hemp inflorescences (sample C3) with the DVB/CAR/PDMS fibre at 40 °C: (**a**) equilibrium and extraction time of 20 and 10 min, respectively; (**b**) equilibrium and extraction time of 30 and 20 min, respectively.

For the outcome of the overall extraction procedure, it is also important to properly set the extraction temperature, together with the equilibrium time and the extraction time. On the basis of previous data available in the literature [14,15,27,28], temperatures of 40, 60, and 80 °C, equilibrium times of 20 and 30 min and extraction times of 10 and 20 min were evaluated in this study. As concerns the effect of temperature, a lower temperature was found to be much better, since an increase in the extraction temperature led to a loss in monoterpenes with respect to the sesquiterpenes. In general, the chromatographic profile of hemp volatile compounds at 40 °C was characterised by higher peak areas (Figure 9); therefore, it was finally selected as the optimal extraction temperature. Being the sample submitted to a relatively low temperature, longer equilibrium and extraction times are usually required for a satisfactory recovery of volatile compounds; indeed, higher peak areas were obtained from hemp when an equilibrium time and an extraction time of 30 and 20 min, respectively, were applied (Figure 10).

**Figure 11:**
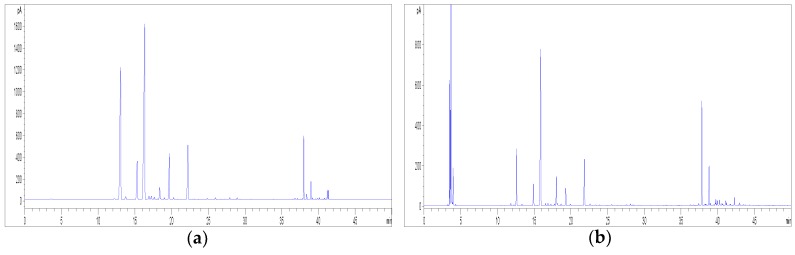
(**a**) GC-FID chromatogram of hemp inflorescences obtained by applying the HS-SPME procedure optimized (sample C3); (**b**) GC-FID chromatogram of the essential oil of hemp sample C3.

In Figure 11, a representative GC-FID chromatogram obtained with the final HS-SPME conditions is shown, reflecting the profile of the essential oil distilled from the same plant material.

### 2.8. GC Methods for the Analysis of Volatile Compounds from Hemp 

**Figure 12:**
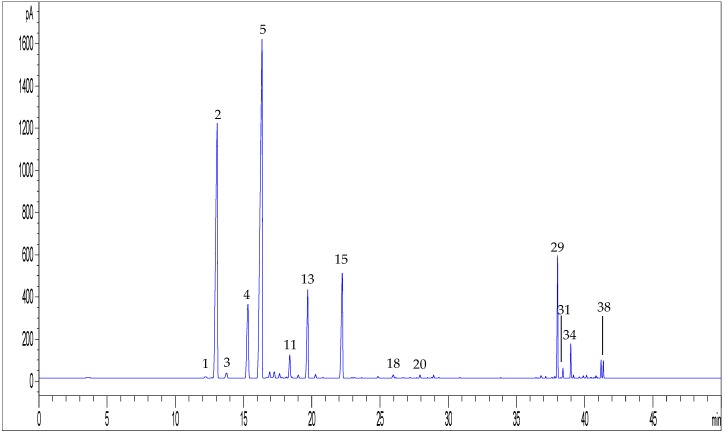
Representative GC-FID chromatogram obtained by using the HS-SPME method optimised for volatile compounds from hemp inflorescences (sample C3). For peak numbering, see Table 4.

Hemp volatile compounds extracted by HS-SPME were analysed by GC-FID and GC-MS and they were identified according to both their calculated linear retention index (*LRI*) and MS spectra, which were compared with available data in the literature and MS spectral libraries, respectively. As shown in Figure 12 and Table 4, the GC analysis allowed us to identify 44 compounds in hemp inflorescences. 

In general, all the hemp samples analysed in this study displayed the same qualitative and semi-quantitative profile: monoterpenes were the most represented class of compounds among volatiles, ranging between 47% and 89% of the total peak area. Sample C5 was the only exception to this trend, as the total area of sesquiterpenes (52% of the total area) was found to be higher than that of monoterpenes. 

β-Myrcene, α- and β-pinene, and limonene represented the most abundant compounds among monoterpenes. It is worthy of notice that the head space of sample C2 had a remarkably high relative content of α-pinene, while samples C4 and C6 displayed a notably high relative amount of limonene.

As to the sesquiterpenes, β-caryophyllene was the most abundant compound in all the samples analysed, followed by α-humulene. Curiously, sample C5 was the one with the higher relative content of caryophyllene oxide, followed by sample C2. It should be pointed out that samples C2 and C5 were the same in that had a cannabinoid profile opposite to that of fresh plant material, further suggesting their aging or non-optimal storage conditions. 

Nevertheless, the GC data of the hemp inflorescences analysed in this work are in accordance with the available one previously described in the literature relating to hemp essential oil or volatile fraction [14,15,27,28,29].

## 3. Materials and Methods 

### 3.1. Chemicals and Solvents

Cannabidiolic acid (CBDA), cannabigerolic acid (CBGA), cannabigerol (CBG), and cannabidiol (CBD) standard solutions (1 mg/mL in methanol or acetonitrile) were purchased from Cerilliant (Round Rock, TX, USA). Chrysoeriol was purchased from Sigma-Aldrich (Milan, Italy), while canniprene was kindly provided by Dr. Federica Pollastro of the Department of Drug Sciences of the University of Piemonte Orientale (Novara, Italy). Formic acid (HCOOH) and HPLC-grade solvents, including EtOH, MeOH, ACN, acetone, EtOAc, *n-*hexane, DCM, ethyl ether and toluene, were from VWR (Milan, Italy). H_2_O was purified by using a Milli-Q Plus185 system from Millipore (Milford, MA, USA). 

### 3.2. Hemp Plant Material

Six samples of fibre-type hemp female inflorescences (indicated in the text as C1–C6, respectively) were analysed in this study. including Antal (C1), Carma (C2), Carmagnola (C3), China (C4), Codimono (C5), and Fibrante (C6). These samples (about 100–500 g dry material each), belonging to different breeding lines, were cultivated under the same agronomic conditions; they were kindly provided by Dr. Gianpaolo Grassi of the research centre CREA-CIN (Rovigo, Italy) and they were certified for a content of Δ^9^-THC below 0.2% (w/w). All the samples considered in this study were approved for commercial use by the European Union [6].

The “Fibrante” sample (C6) was selected for the HPLC method development. The sample “Carmagnola” (C3) was used for the optimisation of the HS-SPME procedure; an essential oil sample of the same hemp variety, obtained by hydro-distillation, was also available for a comparison. 

For each sample, hemp inflorescences were manually separated from twigs and seeds. After this procedure, the samples were stored at +4.0 °C until required for chemical analysis. Hemp inflorescences were ground in a mortar before the extraction.

### 3.3. Sample Preparation

#### 3.3.1. Extraction of Non-Psychoactive Cannabinoids 

Cannabinoids were extracted from hemp inflorescences by means of DM [9]. Briefly, a weighed amount of sample (0.25 g) was extracted with 10 mL of EtOH as the extraction solvent at room temperature for 15 min, under magnetic stirring. The solution was then paper filtered and the residue was extracted with the same procedure twice more with 10 and 5 mL of solvent, respectively. The filtrates of the three extractions were then combined and adjusted to 25 mL with the solvent in a volumetric flask. Before the injection in the HPLC system, the extracts were filtered by using a 0.45 µm PTFE filter into a HPLC vial.

The extraction procedure was repeated twice for each sample.

#### 3.3.2. Extraction of Flavonoids and Related Compounds 

A portion of 0.25 g of hemp inflorescences was treated with 10 mL of *n*-hexane at room temperature for 15 min, under magnetic stirring, in order to remove non-psychoactive cannabinoids. The solution was then filtered on a paper filter and the filtrate sent to waste. The same procedure was repeated twice more, by adding 10 and 5 mL of solvent to the residue, respectively. The residue was then let to dry and, subsequently, it was extracted with 10 mL of acetone. The solution was then filtered on a paper filter into a volumetric flask and the residue was extracted with the same procedure twice more with 10 and 5 mL of solvent, respectively. The filtrates of the three extractions were then pooled, concentrated under vacuum at 30 °C, and adjusted to a final volume of 1 mL with the extraction solvent. Before the injection into the HPLC system, the extracts were filtered by using a 0.45 µm PTFE filter into a HPLC vial.

The extraction procedure was repeated twice for each sample.

#### 3.3.3. Extraction of Volatile Compounds 

HS-SPME was used for hemp volatiles. To do this, a manual holder and a 1 cm stable-flex 50/30 μm DVB/CAR/PDMS fibre were employed (Supelco, Bellefonte, PA, USA). Before GC analysis, the fibre was conditioned in the injector, according to the instructions provided by the manufacturer.

A 400 mg amount of hemp inflorescences, previously ground, was placed in a 10 mL flat-bottom headspace vial sealed with a magnetic crimp cap and PTFE/silicone septa (Supelco). Under the optimized conditions, the sample was heated for 30 min during the equilibrium time in a thermostatic bath at 40 °C. The SPME device was then inserted into the sealed vial by manually penetrating the septum and the fibre was exposed to the headspace for 20 min during the extraction time. After sampling, the SPME fibre was immediately inserted into the GC injector and thermally desorbed. A desorption time of 5 min at 250 °C was used in the split-less mode. Before sampling, the fibre was reconditioned for 5 min in the GC injector port at 250 °C.

The extraction procedure was repeated three times for each sample.

### 3.4. HPLC-UV/DAD Conditions 

The HPLC-UV/DAD analyses were performed on an Agilent Technologies (Waldbronn, Germany) modular model 1100 system, consisting of a vacuum degasser, a quaternary pump, an autosampler, a thermostated column compartment and a diode array detector (UV/DAD). Chromatograms were recorded by using an Agilent Chemstation for LC and LC-MS systems (Rev. B.01.03).

#### 3.4.1. HPLC-UV/DAD Analysis of Non-Psychoactive Cannabinoids

The chromatographic conditions for the analysis of non-psychoactive cannabinoids were set on the basis of a previous work [9]. HPLC analyses were carried out on an Ascentis Express C_18_ column (150 mm × 3.0 mm I.D., 2.7 μm, Supelco, Bellefonte, PA, USA), with a mobile phase composed of 0.1% HCOOH in both (A) H_2_O and (B) ACN. The gradient elution was modified as follows: 0–13 min 60% B, 13–17 min from 60% to 80% B, 17–22 min from 80% to 90% B. The post-running time was 15 min. The flow-rate was 0.4 mL/min. The column temperature was set at 30 °C. The sample injection volume was 3 µL. The UV/DAD acquisitions were carried out in the range 190–600 nm, while chromatograms were acquired at 210 nm (for decarboxylated cannabinoids) and at 220 nm (for cannabinoic acids) [9]. Three injections were performed for each sample.

#### 3.4.2. HPLC-UV/DAD Analysis of Flavonoids and Related Compounds

Regarding the analysis of flavonoids and related compounds, the chromatographic column and the mobile phase were the same as those used for the analysis of cannabinoids. Conversely, the gradient elution was optimized as follows: 0–5 min 40% B, 5–20 min from 40 to 80% B; 20–35 min from 80% to 90% B, which was held for 10 min. The post-running time was 10 min. The flow rate and the sample injection volume were kept at 0.4 mL/min and 3 μL, respectively and column was kept at room temperature. As regards detection, UV/DAD acquisitions were carried out in the range 190–600 nm, while chromatograms were acquired at 210 nm (for canniprene) and at 342 nm (for cannflavins). Three injections were performed for each sample.

### 3.5. HPLC-ESI-MS and MS^2^ Conditions

The HPLC-ESI-MS and MS^2^ analyses were performed on an Agilent Technologies modular 1200 system, equipped with a vacuum degasser, a binary pump, a thermostated autosampler, a thermostated column compartment, and a 6310A ion trap mass analyser with an ESI ion source. The HPLC column and the applied chromatographic conditions were the same as those reported for the HPLC-UV/DAD systems. In details, the chromatographic conditions described in Section 3.4.1 and Section 3.4.2 were applied for the HPLC-ESI-MS and MS^2^ experiments of cannabinoids and polyphenols, respectively.

The HPLC-ESI-MS system was operated both in the positive and in the negative ion mode. For the positive ion mode, the experimental parameters were set as follows: the capillary voltage was 3.5 kV, the nebulizer (N_2_) pressure was 32 psi, the drying gas temperature was 350 °C, the drying gas flow was 10 L/min, and the skimmer voltage was 40 V. For the negative ion mode, the MS conditions were the same as described above.

Data were acquired by Agilent 6300 Series Ion Trap LC/MS system software (version 6.2). The mass spectrometer was operated in the full-scan mode in the *m*/*z* range 200–1200. MS^2^ spectra were automatically performed with helium as the collision gas in the *m*/*z* range 50–1500, by using the SmartFrag function, which automatically selects the precursor ion to be fragmented and it eliminates the need for a time-consuming manual collision-induced dissociation (CID) voltage optimization [9].

### 3.6. HPLC-UV/DAD Method Validation

In this study, the validation of the HPLC-UV/DAD method for the determination of hemp flavonoids and related compounds was carried out, since the chromatographic method for the quantification of non-psychoactive cannabinoids was previously fully validated [9]. The validation was performed in agreement with the international guidelines for analytical techniques for the quality control of pharmaceuticals (ICH guidelines) [35]. 

As regards linearity, the stock standard solution of non-psychoactive cannabinoids (CBDA, CBGA, CBG and CBD) was prepared as follows: an accurately measured volume of reference solution (100–200 μL) was placed into a 1 mL volumetric flask; then, MeOH was added and the solution was diluted to volume with the same solvent. As to flavonoids, the stock standard solution of each compound (canniprene and chrysoeriol) was prepared by weighing an accurate amount of reference compound (0.9 mg) into a 1 mL volumetric flask; then, the solution was adjusted to volume with MeOH. The external standard calibration curve was generated by using six data points, covering the concentration ranges: 2.5–200.0 µg/mL for CBDA, CBGA and CBD; 1.3–100.0 µg/mL for CBG; 0.3–23.4 µg/mL for canniprene, and 1.3–43.0 µg/mL for chrysoeriol. Three µL aliquots of each standard solution were used for HPLC analysis. Injections were performed in triplicate for each concentration level. The calibration curve was obtained by plotting the peak area of the compound at each level versus the concentration of the sample. The quantification of non-psychoactive cannabinoids and canniprene was performed by using their calibration curves. The amount of cannflavins A and B in hemp samples was determined by using the calibration curve of chrysoeriol, having the same chromophore, and the content was corrected by using the molecular weight ratio.

For reference compounds, the limit of detection (LOD) and the limit of quantification (LOQ) were experimentally determined by HPLC analysis of serial dilutions of a standard solution to reach a signal-to-noise (*S*/*N*) ratio of 3 and 10, respectively.

The precision of the extraction technique was validated by repeating the extraction procedure on the inflorescence of the same hemp sample (C6) six times. An aliquot of each extract was then injected and quantified. The precision of the chromatographic system was tested by performing intra- and inter-day multiple injections of one extract from sample C6 and then checking the %RSD of retention times and peak areas. Six injections were performed each day for three consecutive days. 

### 3.7. GC-FID Analysis of Volatile Compounds

These analyses were performed on an Agilent Technologies 7820 gas chromatograph with a flame ionization detector (FID). Compounds were separated on an Agilent Technologies HP-5 cross-linked poly-5% diphenyl-95% dimethyl polysiloxane (30 m × 0.32 mm i.d., 0.25 µm film thickness) capillary column. The column temperature was initially set at 45 °C, then increased to 100 °C at a rate of 2 °C/min up, then raised to 250 °C at a rate of 5 °C/min, which was held for 5 min. Helium was used as the carrier gas, at a flow rate of 1.0 mL/min. The injector and detector temperature was set at 250 and 300 °C, respectively.

### 3.8. GC-MS Analysis of Volatile Compounds

These analyses were carried out on a 7890A gas chromatograph coupled with a 5975C network mass spectrometer (Agilent Technologies, Germany). Compounds were separated on an Agilent Technologies HP-5 MS cross-linked poly-5% diphenyl-95% dimethyl polysiloxane (30 m × 0.25 mm i.d., 1.00 μm film thickness) capillary column. The temperature program was the same as described above. Helium was used as the carrier gas, at a flow rate of 0.7 mL/min. The injector, transfer line and ion-source temperature was 250, 280, and 230 °C, respectively. MS detection was performed with electron ionization (EI) at 70 eV, operating in the full-scan acquisition mode in the *m*/*z* range 40–400.

### 3.9. Qualitative and Semi-Quantitative Analysis of Volatile Compounds

Hemp volatile compounds were identified by comparing the retention times of the chromatographic peaks with those of authentic reference standards run under the same conditions and by comparing with the literature the linear retention index (*LRI*) relative to a mixture of *n*-alkanes (C_8_–C_40_) in *n*-hexane loaded onto the DVB-CAR-PDMS fibre and injected under the same conditions. Peak enrichment by co-injection with authentic reference compounds was also carried out. Comparison of the MS-fragmentation pattern of the target analytes with those of pure components was performed. A mass-spectrum database search was performed by using the National Institute of Standards and Technology (NIST, Gaithersburg, MD, USA) mass-spectral database (version 2.0d, 2005). The percentage relative amount of individual components was expressed as percent peak area relative to total peak area. 

## 4. Conclusions

A comprehensive analysis of the bioactive compounds in hemp inflorescences was carried out in this research work for the first time. The chemical classes considered in this study encompassed non-psychoactive cannabinoids, flavonoids and related compounds, and volatile compounds.

In detail, the qualitative and quantitative profiling of non-psychoactive cannabinoids was performed by applying an HPLC method with UV/DAD, ESI-MS and MS^2^ detection. A new HPLC method with UV/DAD, ESI-MS and MS^2^ detection was developed and validated for the analysis of hemp-specific flavonoids, combined with a selective and simple extraction procedure. Finally, the characterisation of the hemp volatile fraction was achieved by optimising a new HS-SPME-GC procedure with FID and MS detection. 

All the analytical methods developed in the present work clearly demonstrated the provision of a comprehensive and reliable analysis of the bioactive compounds present in hemp inflorescences, which are known to be characterised by a very complex composition. Therefore, they can be applied for a detailed chemical characterisation of the plant material in order to monitor its composition and guarantee a better reproducibility of biological assays, since several compounds may act synergistically, and also in quality control to ensure the efficacy and safety of hemp-based products to be used in the pharmaceutical and nutraceutical fields.

## Figures and Tables

**Table 1:** Retention times, UV, MS, and MS^2^ data of the main non-psychoactive cannabinoids, flavonoids and related compounds in hemp extracts.

Peak Number	Compound	*t*_R_ (min)	UV λ*_max_* (nm)	MS (*m*/*z*) ^a^	MS^2^ (*m*/*z*) ^a^	MS (*m*/*z*) ^b^	MS^2^ (*m*/*z*) ^b^
1	CBDA	13.8	225,269,307	359, 341 *	219 (100), 261 (55), 285 (33), 232 (22)	357	339 (100), 313 (9)
2	CBGA	15.7	223,268,305	361, 343 *	219 (100), 261 (49), 233 (19)	359	341 (100), 359 (6)
3	CBG	17.1	207,231sh,273	317	207 (100), 233 (40), 280 (10)	315	271 (100), 204 (80), 247 (51), 297 (49)
4	CBD	17.9	209,232sh,276	315	259 (100), 233 (26), 221 (24), 207 (24)	313	245 (100), 210 (6)
5	CFL-B	14.5	215,273,342	370	313 (100)	-	-
6	Canniprene	16.9	204,280	343, 287 *	255 (100), 227 (98), 269 (11)	341	326 (100), 269 (84), 283 (43), 257 (12)
7	CFL-A	21.1	214,274,342	437	313 (100)	-	-

Experimental conditions as described in Section 3.4 and Section 3.5. ^a^ Positive ion mode. ^b^ Negative ion mode. * Precursor ion.

**Table 2:** Amount (mg/g) of the main non-psychoactive cannabinoids in hemp inflorescences ^a^.

Compound	C1	C2	C3	C4	C5	C6
CBDA	22.6 ± 2.5	4.1 ± 1.7	36.4 ± 1.0	4.1 ± 0.1	3.7 ± 0.2	17.6 ± 0.4
CBGA	0.4 ^b^	0.2 ± 0.1	0.6 ^b^	0.2 ^b^	<LOQ	0.1 ^b^
CBG	<LOQ	0.3 ± 0.1	<LOQ	0.3 ^b^	<LOQ	<LOQ
CBD	1.9 ± 0.2	5.9 ± 0.4	6.3 ± 0.3	5.9 ± 0.1	8.6 ± 0.5	6.4 ± 0.4

Experimental conditions as in Section 3.4. ^a^ Data are expressed as mean (*n* = 6) ± SD. ^b^ SD < 0.05.

**Table 3:** Amount (µg/g) of the main flavonoids and related compounds in hemp inflorescences ^a^.

Compound	C1	C2	C3	C4	C5	C6
CFL-A	163.4 ± 2.4	109.4 ± 5.7	318.9 ± 5.8	59.9 ± 3.4	89.5 ± 6.7	140.6 ± 12.5
CFL-B	100.9 ± 1.7	21.0 ± 2.6	412.2 ± 4.0	25.3 ± 0.2	61.4 ± 5.7	75.9 ± 6.6
Canniprene	<LOD	<LOD	<LOD	<LOD	<LOD	<LOD

Experimental conditions as in Section 3.4. ^a^ Data are expressed as mean (*n* = 6) ± SD. For sample C6, *n* = 18.

**Table 4:** Semi-quantitative data (% relative peak area values) for volatile compounds from hemp inflorescences ^a^.

Peak Number	Compound	*LRI* Calc.	*LRI* Lit. ^c^	% Relative Peak Area					
				C1	C2	C3	C4	C5	C6
1	α-Thujene	932	911	3.7 ± 1.1	1.4 ± 1.0	0.2 ^b^	0.2 ^b^	0.5 ± 0.1	-
2	α-Pinene	939	932	20.1 ± 0.5	40.1 ± 2.3	26.7 ^b^	9.6 ± 0.4	15.9 ± 2.1	10.3 ^b^
3	Camphene	952	957	0.3 ^b^	-	0.5 ^b^	0.3 ^b^	0.3 ^b^	0.2 ^b^
4	β-Pinene	980	978	5.7 ^b^	9.3 ± 0.1	6.9 ± 0.1	2.9 ± 0.2	4.2 ± 0.3	3.7 ^b^
5	β-Myrcene	1000	992	28.7 ± 0.1	12.9 ± 0.1	36.9 ± 0.2	32.1 ± 3.3	6.9 ± 0.5	34.1 ± 1.1
6	α-Phellandrene	1009	1007	0.1 ^b^	-	0.4 ^b^	0.5 ^b^	-	0.3 ^b^
7	Δ^3^-Carene	1014	1010	1.3 ^b^	1.4 ^b^	0.5 ^b^	0.6 ^b^	-	0.3 ^b^
8	α-Terpinene	1020	1018	-	-	0.3 ^b^	0.4 ^b^	-	0.3 ± 0.1
9	*o*-Cymene	1022	1021	0.1 ^b^	-	0.1 ^b^	0.1 ^b^	1.3 ± 0.1	-
10	*p*-Cymene	1028	1026	0.2 ^b^	-	0.1 ^b^	0.5 ± 0.1	2.5 ± 0.1	0.8 ± 0.1
11	Limonene	1032	1035	2.4 ^b^	2.3 ± 0.1	1.6 ^b^	11.3 ± 0.5	3.5 ± 0.2	11.5 ± 0.3
12	*cis*-Ocimene	1042	1040	0.5 ^b^	0.7 ^b^	0.2 ^b^	0.6 ± 0.1	0.4 ± 0.1	-
13	*trans*-Ocimene	1053	1097	7.1 ± 0.3	0.6 ^b^	6.1 ± 0.2	5.2 ± 0.1	0.4 ± 0.1	0.5 ^b^
14	γ-Terpinene	1062	1062	0.2 ^b^	-	0.3 ^b^	0.4 ^b^	0.3 ^b^	0.5 ^b^
15	α-Terpinolene	1093	1088	3.9 ± 0.1	-	7.9 ± 0.1	12.0 ± 0.8	1.4 ± 0.1	4.9 ± 0.1
16	Linalool	1104	1104	0.6 ^b^	-	0.1 ^b^	0.3 ^b^	0.8 ± 0.1	0.4 ^b^
17	6-Camphenol	1106	1110	0.3 ^b^	-	-	0.1 ^b^	1.2 ± 0.1	-
18	neo-Alloocimene	1133	1143	0.4 ^b^	-	0.1 ^b^	0.4 ^b^	1.1 ± 0.2	-
19	Isopinocarveol	1145	1139	-	0.6 ^b^	-	-	0.6 ± 0.1	-
20	Terpinen-4-ol	1181	1177	0.5 ^b^	-	0.2 ^b^	0.3 ^b^	3.3 ± 0.2	0.6 ^b^
21	*p*-Cymen-8-ol	1183	1181	-	-	0.1 ^b^	-	2.2 ± 0.2	-
22	α-Terpineol	1193	1195	0.1 ^b^	-	-	0.1 ^b^	0.7 ± 0.1	0.4 ^b^
23	Eugenol	1371	1373	-	-	-	0.3 ± 0.1	-	-
24	α-Copaene	1375	1376	-	-	-	0.8 ± 0.2	-	0.3 ± 0.1
25	Geranyl acetate	1379	1386	-	-	-	0.3 ± 0.1	-	-
26	Ylangene	1389	1406	-	-	0.1 ^b^	-	-	-
27	*Z*,*Z*-α-Farnesene	1415	1462	0.2 ^b^	0.7 ^b^	-	0.1 ^b^	1.2 ^b^	0.4 ^b^
28	α-Santalene	1423	1420	-	-	0.1 ^b^	0.1 ^b^	0.3 ^b^	-
29	β-Caryopyllene	1430	1428	13.4 ± 0.1	18.1 ± 0.5	5.2 ± 0.1	8.1 ± 2.0	22.6 ± 1.0	21.8 ± 0.7
30	*Z*,*E*-α-Farnesene	1437	1486	-	-	-	0.1 ^b^	2.2 ^b^	-
31	α-Guaiene	1444	1439	0.2 ^b^	0.5 ^b^	0.4 ^b^	0.9 ± 0.3	-	-
32	Aromadendrene	1446	1449	0.4 ± 0.1	-	-	0.3 ± 0.1	-	-
33	β-Farnesene	1460	1454	0.1 ^b^	-	-	1.8 ± 0.5	0.3 ^b^	-
34	α-Humulene	1464	1455	2.8 ^b^	4.6 ± 0.3	1.3 ^b^	2.1 ± 0.5	8.7 ± 0.6	5.6 ± 0.2
35	Alloaromadendrene	1471	1467	0.3 ^b^	0.6 ^b^	0.1 ^b^	0.3 ± 0.1	1.4 ± 0.2	0.2 ^b^
36	Germacrene D	1489	1480	-	-	-	0.1 ^b^	0.5 ^b^	-
37	β-Selinene	1493	1485	0.3 ^b^	0.6 ^b^	-	0.2 ^b^	0.5 ± 0.1	0.1 ^b^
38	Valencene	1496	1496	0.5 ^b^	-	0.1 ^b^	0.1 ^b^	1.4 ± 0.1	0.3 ^b^
39	α-Selinene	1505	1494	0.4 ^b^	0.4 ^b^	0.1 ^b^	0.1 ^b^	1.2 ± 0.1	0.2 ^b^
40	γ-Cadinene	1515	1514	0.3 ^b^	-	-	0.6 ± 0.2	0.4 ^b^	-
41	δ-Cadinene	1532	1530	-	-	0.1 ^b^	0.4 ± 0.1	0.4 ^b^	-
42	α-Calacorene	1547	1542	-	-	-	0.6 ± 0.1	-	0.2 ^b^
43	Celina-3,7(11)-diene	1554	1550	-	-	0.6 ^b^	-	0.3 ^b^	-
44	Caryophyllene oxide	1591	1583	-	0.6 ^b^	0.2 ^b^	0.1 ^b^	2.6 ± 0.4	-
	Total			94.8 ± 0.9	95.4 ± 1.0	97.5 ± 1.3	95.3 ± 1.1	91.5 ± 1.6	97.9 ± 0.7

Experimental conditions as in Section 3.7, Section 3.8 and Section 3.9. ^a^ Data are expressed as mean (*n* = 3) ± SD. ^b^ SD < 0.05. ^c^ *LRI* data from NIST Chemistry WebBook, SRD 69, https://www.nist.gov.

## Supplementary Materials

The Supplementary Materials are available online. Table S1: linearity and sensitivity data for compounds used as hemp standards, Table S2: intra- and inter-day precision data for retention time (*t*_R_) and peak area of the main flavonoids in hemp extracts (sample C6), Table S3: intra- and inter-day precision data for the extraction of the main flavonoids from hemp (sample C6).
