# Supplementary material for: New Methods for the Comprehensive Analysis of Bioactive Compounds in Cannabis sativa L. (hemp)
Source: Molecules. 2018 Oct 14;23(10):2639. doi: 10.3390/molecules23102639 (PMC6222702; doi:10.3390/molecules23102639)
Supplement: Supplementary file 1 [file molecules-23-02639-s001.pdf]

Table S1

Linearity and sensitivity data for compounds used as hemp standards[a]

Compound	Linearity range (μg/mL)	Slope (*a*)	Intercept (*b*)	r^2	LOD (μg/mL)	LOQ (μg/mL)
CBDA	2.5 - 200.0	31.9 ± 0.2	29.6 ± 24.2	0.9996	0.8	2.5
CBGA	2.5 - 200.0	32.4 ± 0.2	30.2 ± 21.4	0.9997	0.8	2.5
CBG	1.3 - 100.0	52.0 ± 0.6	374.3 ± 29.8	0.9990	0.4	1.3
CBD	2.5 - 200.0	74.9 ± 0.8	-60.3 ± 23.3	0.9990	0.8	2.5
Chrysoeriol	1.3 - 43.0	21.4 ± 0.5	29.7 ± 3.6	0.9991	0.4	1.3
Canniprene	0.3 - 23.4	121.2 ± 0.9	81.8 ± 10.2	0.9990	0.1	0.3

Experimental conditions as in Section 3.6.

[a] For each curve the equation is $y = ax + b$, where y is the peak area, x the concentration of the analyte (μg/mL), *a* is the slope, *b* is the intercept and r^2 the correlation coefficient. Standard error (S.E.) values are given in parenthesis. The *p* value was < 0.0001 for all calibration curves.

Table S2

Intra- and inter-day precision data for retention time (t_R) and peak area of the main flavonoids in hemp extracts (sample C6)

	Intra-day precision (*n* = 6, mean)						Inter-day precision (*n* = 18, mean)	
	Day 1		Day 2		Day 3			
	t_R (min) ± RSD (%)	Area (mAU×s) ± RSD (%)	t_R (min) ± RSD (%)	Area (mAU×s) ± RSD (%)	t_R (min) ± RSD (%)	Area (mAU×s) ± RSD (%)	t_R (min) ± RSD (%)	Area (mAU×s) ± RSD (%)
CFL-B	14.6 ± 2.1	516.5 ± 2.5	14.6 ± 1.1	535.1 ± 2.7	14.3 ± 2.4	540.3 ± 2.9	14.5 ± 2.0	530.6 ± 3.2
CFL-A	21.2 ± 1.5	861.7 ± 3.0	21.1 ± 1.2	847.8 ± 1.8	21.0 ± 2.2	860.9 ± 2.9	21.1 ± 1.6	856.8 ± 2.6

Experimental conditions as described in Section 3.6.

Table S3

Intra- and inter-day precision data for the extraction of the main flavonoids from hemp (sample C6)

	Intra-day precision (*n* = 6, mean)			Inter-day precision (*n* = 18, mean)
	Day 1	Day 2	Day 3	
	µg/g ± SD	µg/g ± SD	µg/g ± SD	µg/g ± SD
CFL-B	77.2 ± 11.3	73.2 ± 4.1	77.4 ± 1.3	75.9 ± 6.6
CFL-A	146.9 ± 21.3	137.7 ± 4.1	137.3 ± 4.7	140.6 ± 12.5

Experimental conditions as described in Section 3.6.
